# Supplementary material for: RNF114 and RNF166 exemplify reader-writer E3 ligases that extend K11 polyubiquitin onto sites of MARUbylation
Source: EMBO J. 2025 Oct 2;44(21):5993–6018. doi: 10.1038/s44318-025-00577-z (PMC12583694; doi:10.1038/s44318-025-00577-z)

Exp 420_Input_Tubulin

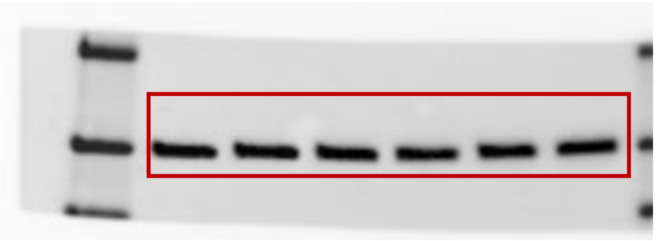

Exp 421_Input_Tubulin

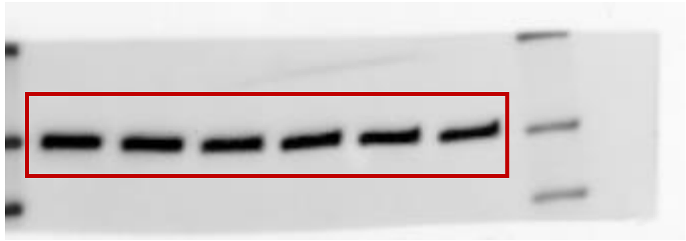

Exp 423_Input_Tubulin

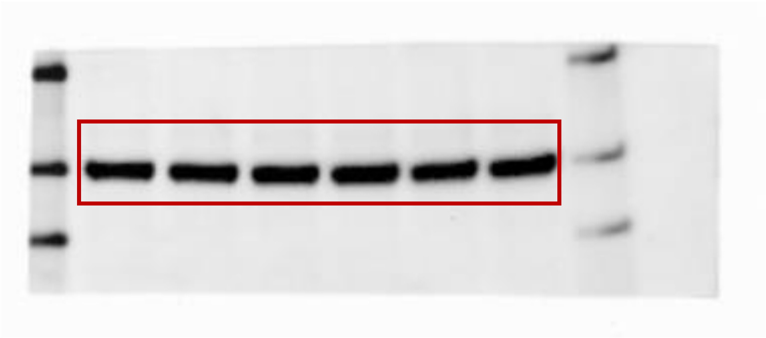

Exp 420_Input_PARP7

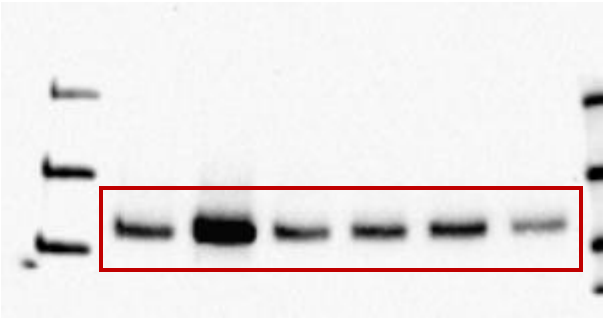

Exp 421_Input_PARP7

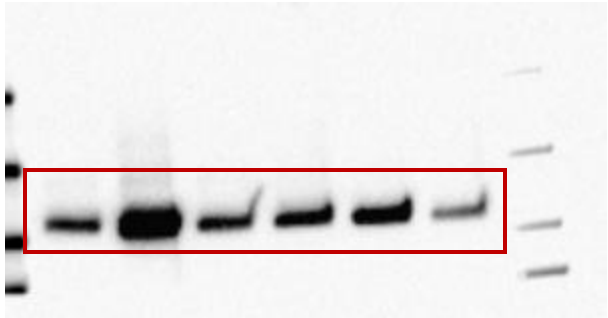

Exp 423_Input_PARP7

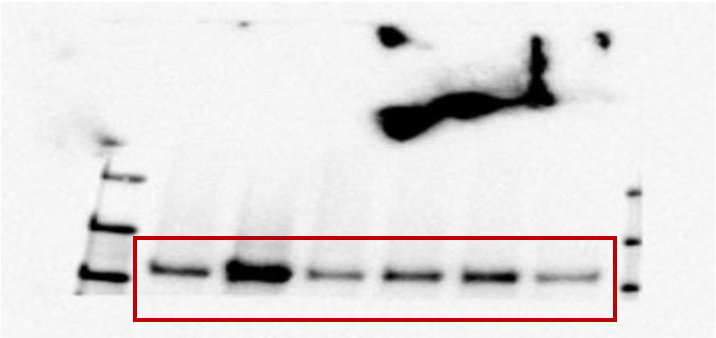

Exp 420_SN_HA_diUB

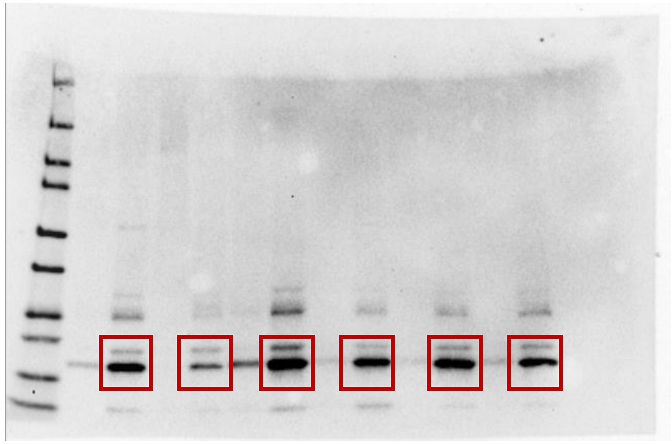

Exp 421_SN_HA_diUB

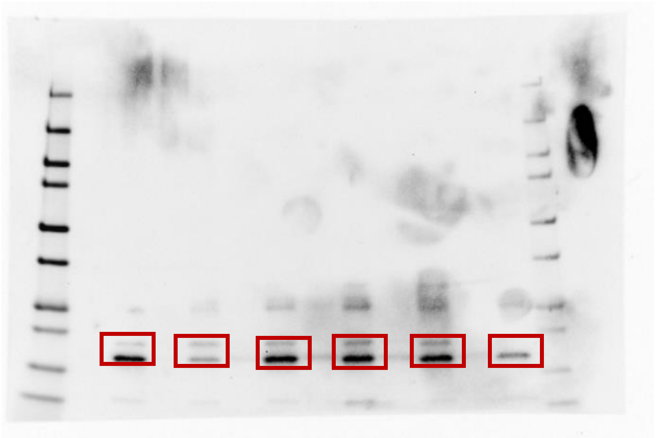

Exp 423_SN_HA_diUB

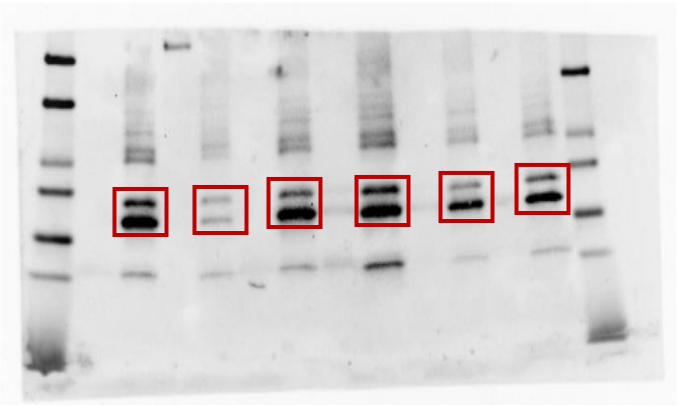

Exp 420_SN_HA_monoUB

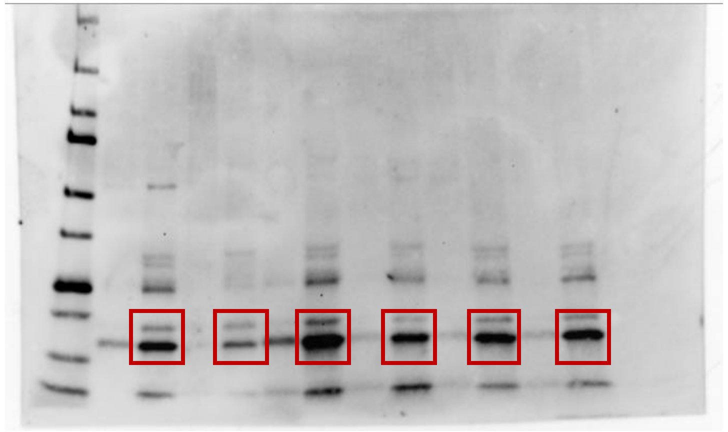

Exp 421_SN_HA_monoUB

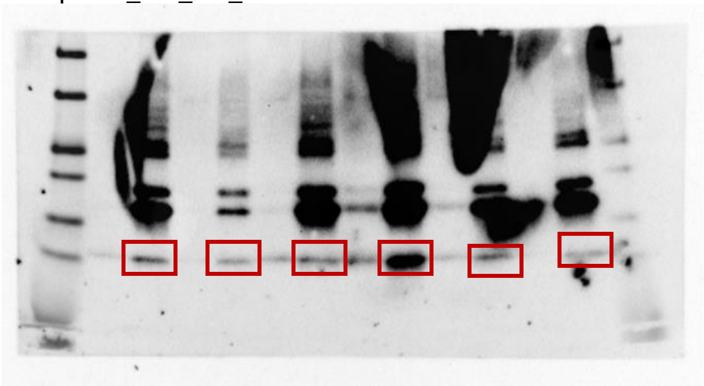

Exp 423_SN_HA_monoUB

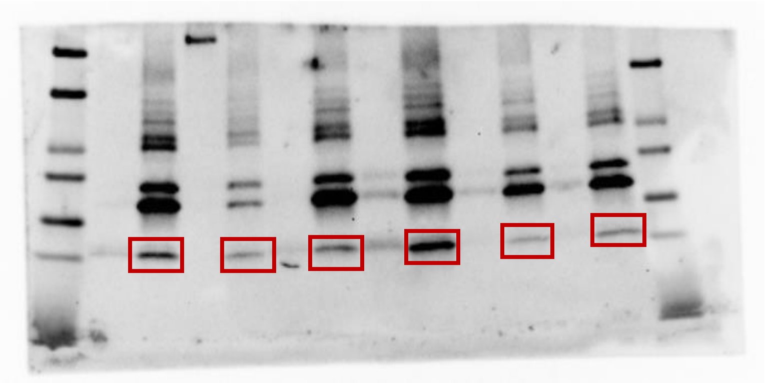

Exp 420_Bound_GFP

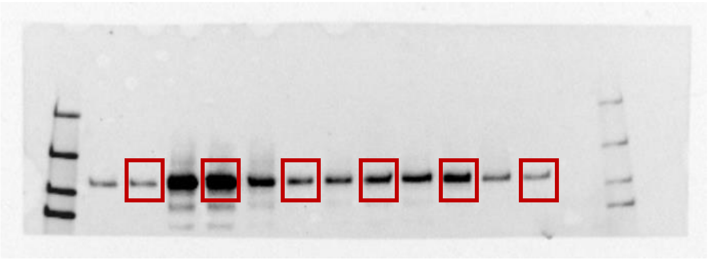

Exp 421_Bound_GFP

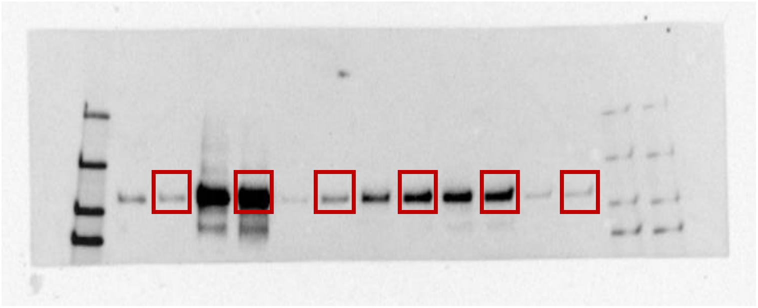

Exp 423_Bound_GFP

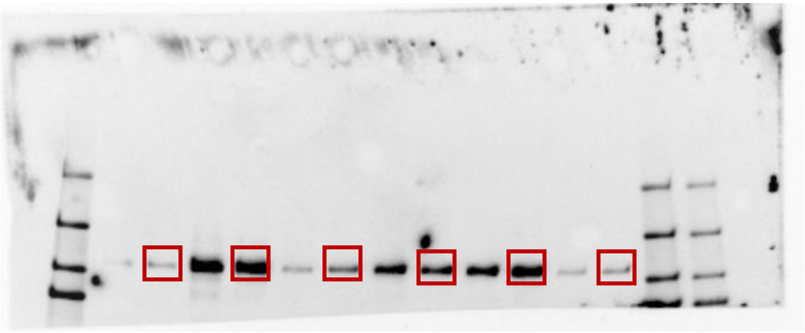

Supplement: Supplementary file 3 — Source data Fig. 1 [file 44318_2025_577_MOESM3_ESM.zip › Figure 1/1C-D/replicate blots/Quantification area-Fig 1E.pdf]
