# Supplementary figures and images for: RNF114 and RNF166 exemplify reader-writer E3 ligases that extend K11 polyubiquitin onto sites of MARUbylation
Source: EMBO J. 2025 Oct 2;44(21):5993–6018. doi: 10.1038/s44318-025-00577-z (PMC12583694; doi:10.1038/s44318-025-00577-z)

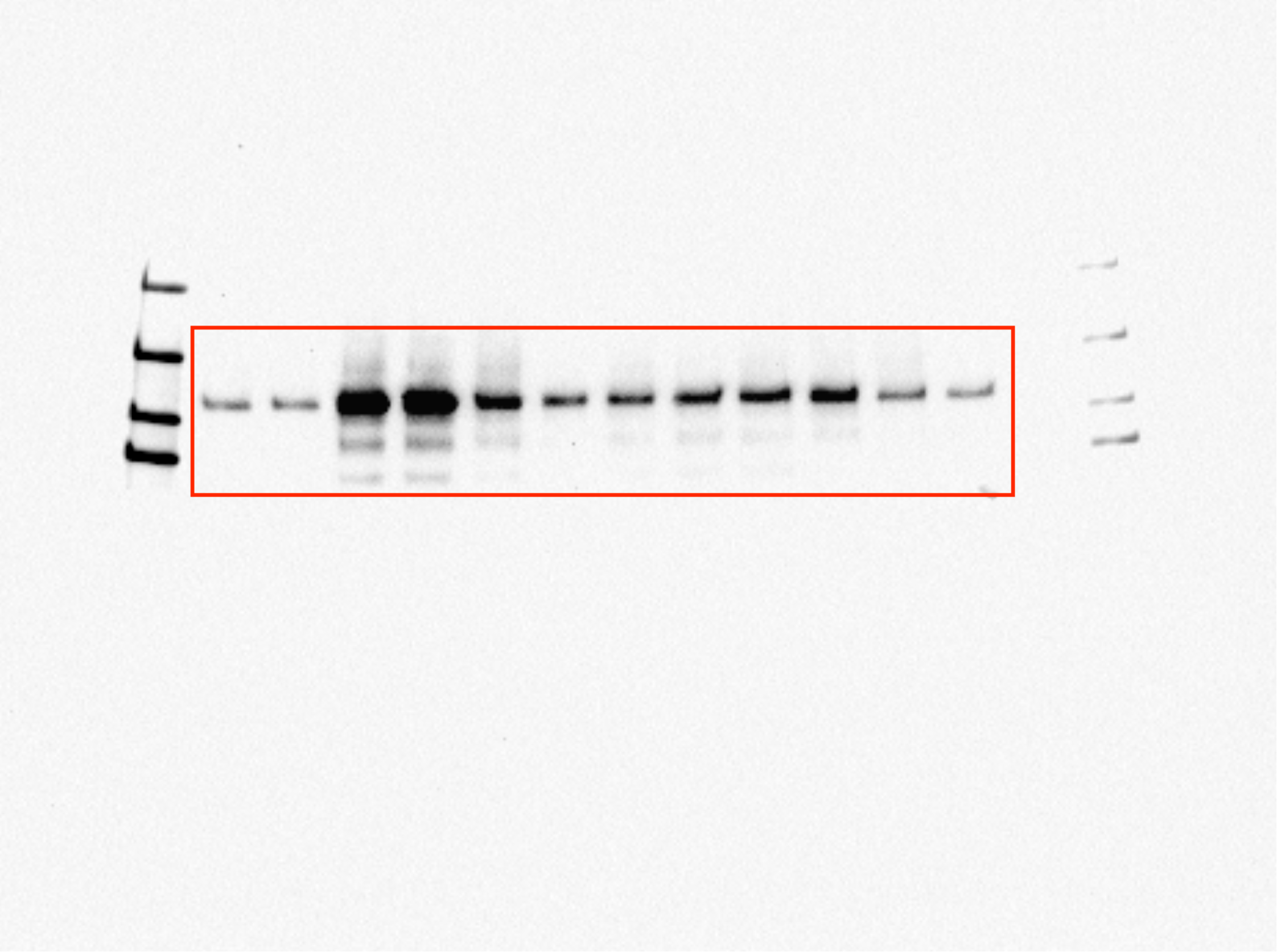

Supplement: Supplementary file 3 — Source data Fig. 1 [file 44318_2025_577_MOESM3_ESM.zip › Figure 1/1C-D/BOUND_GFP.tif]

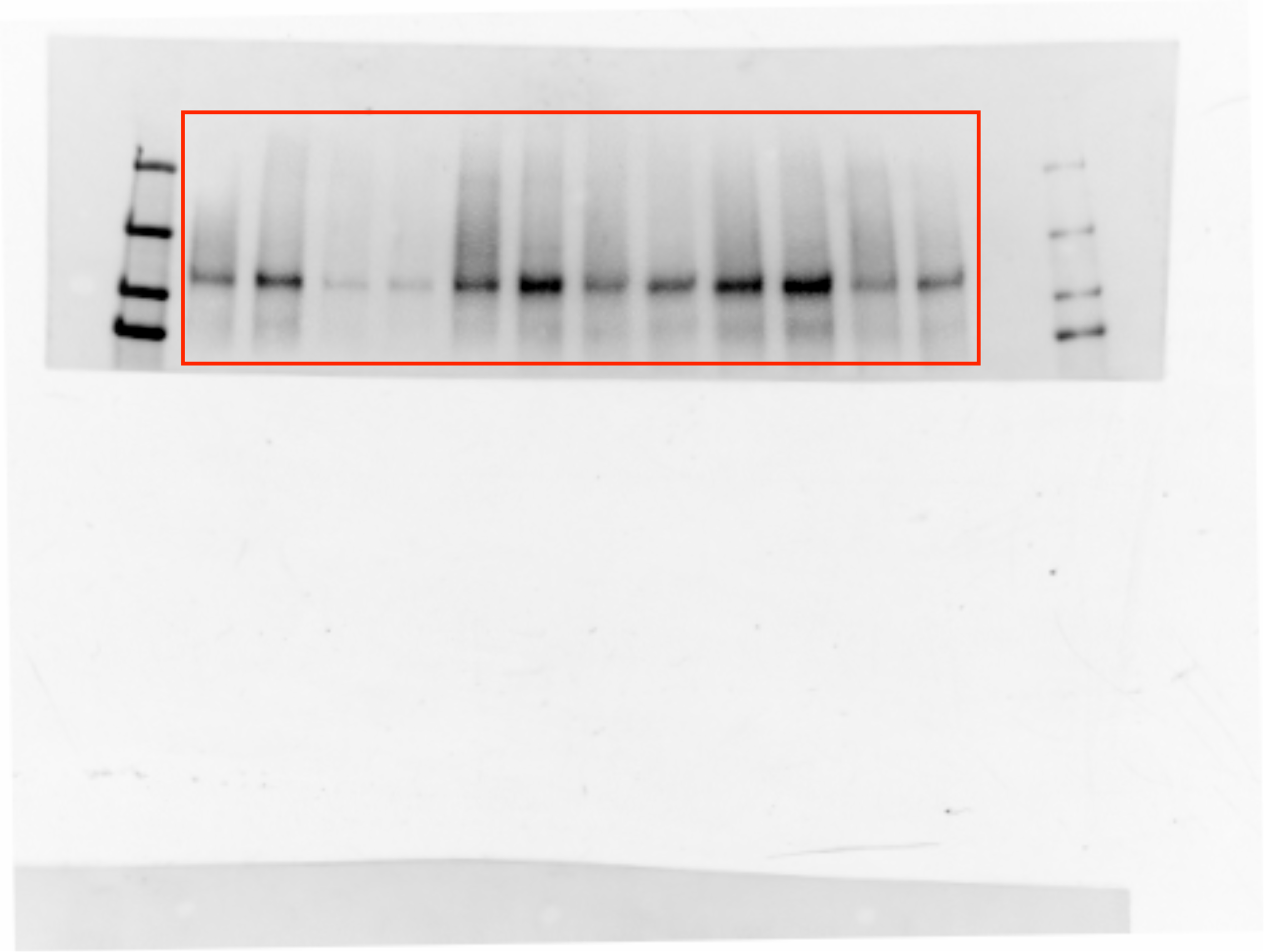

Supplement: Supplementary file 3 — Source data Fig. 1 [file 44318_2025_577_MOESM3_ESM.zip › Figure 1/1C-D/BOUND_33204.tif]

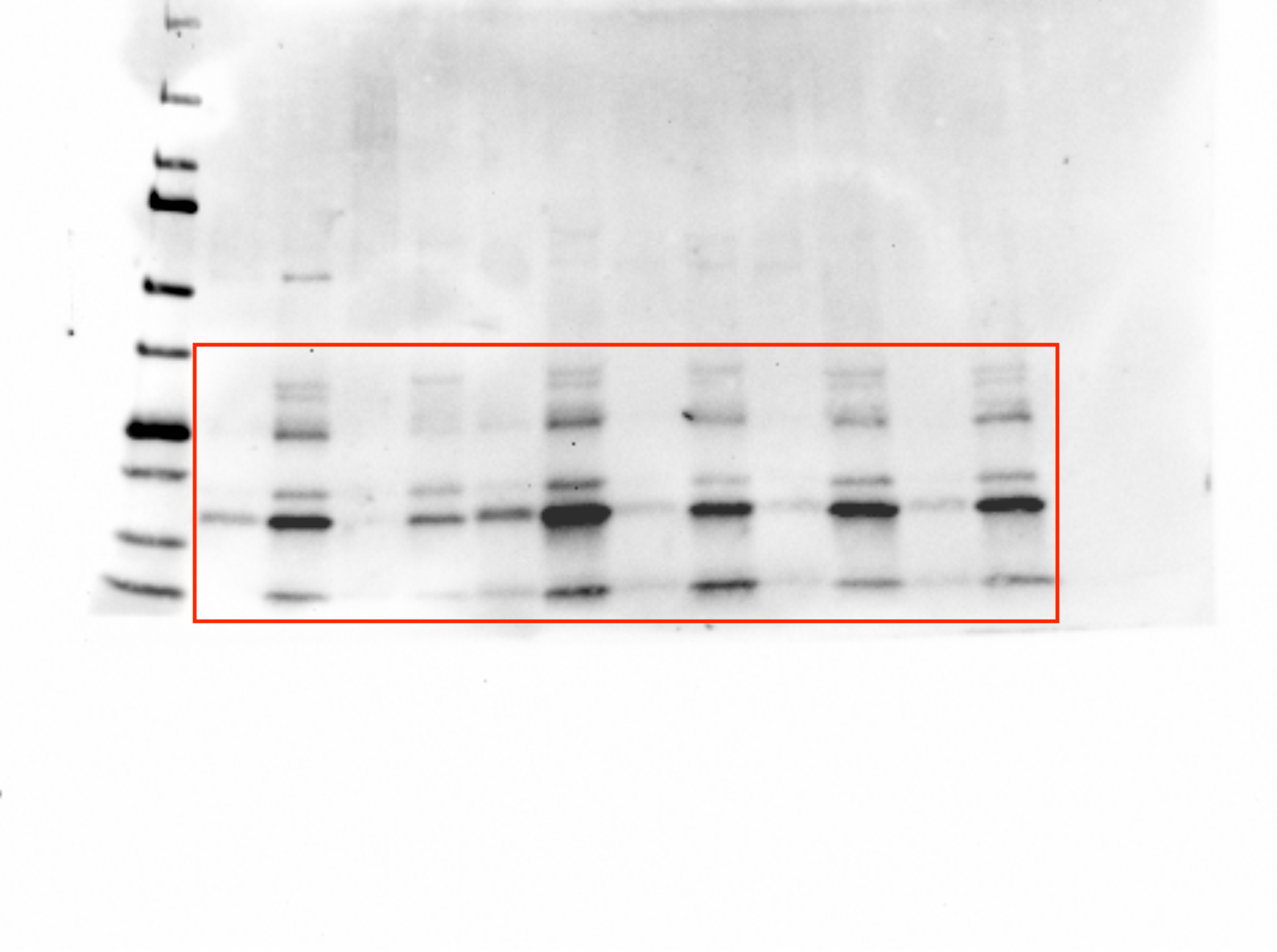

Supplement: Supplementary file 3 — Source data Fig. 1 [file 44318_2025_577_MOESM3_ESM.zip › Figure 1/1C-D/SN_HA_long.tif]

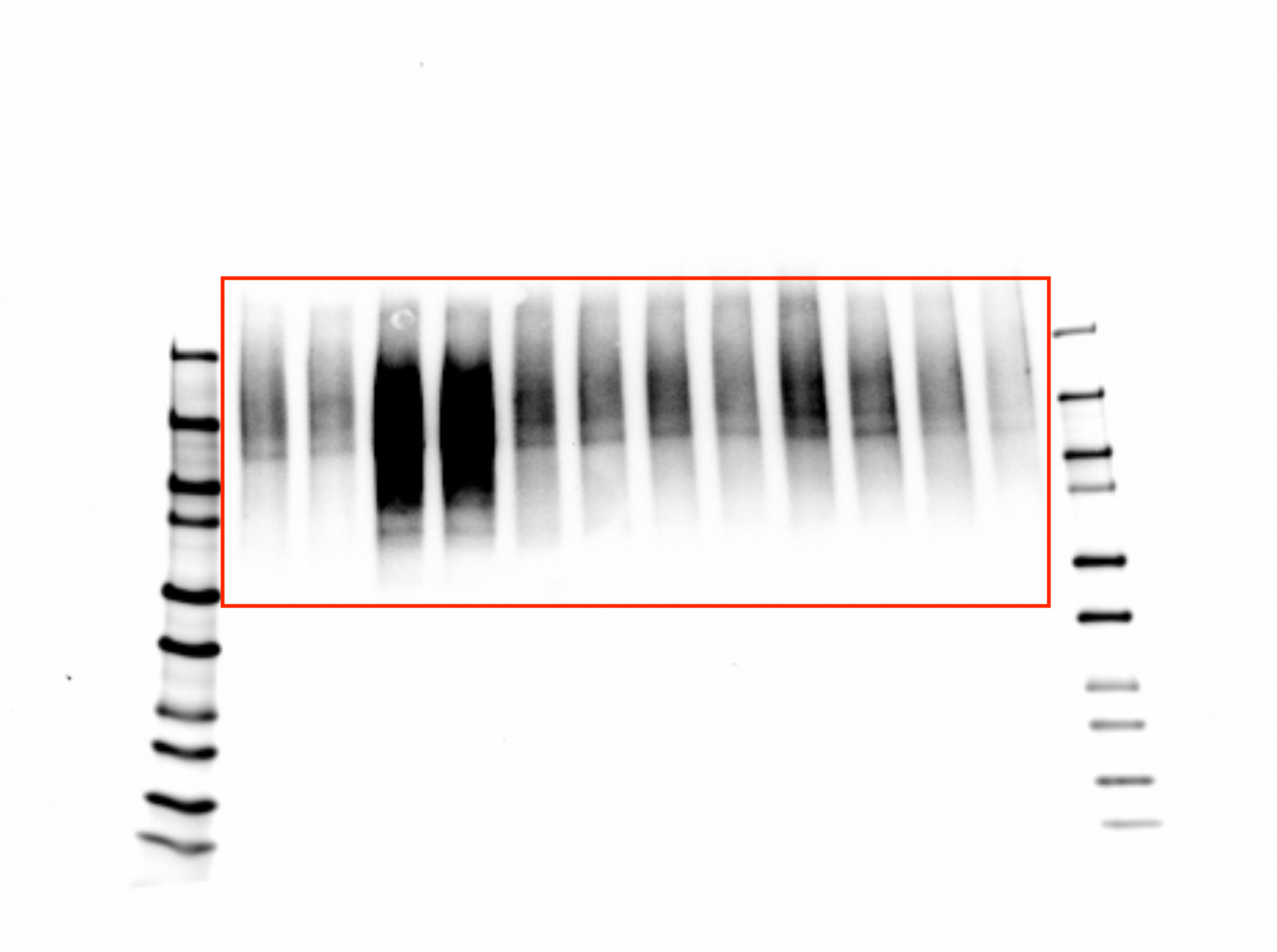

Supplement: Supplementary file 3 — Source data Fig. 1 [file 44318_2025_577_MOESM3_ESM.zip › Figure 1/1C-D/Bound_HA_short.tif]

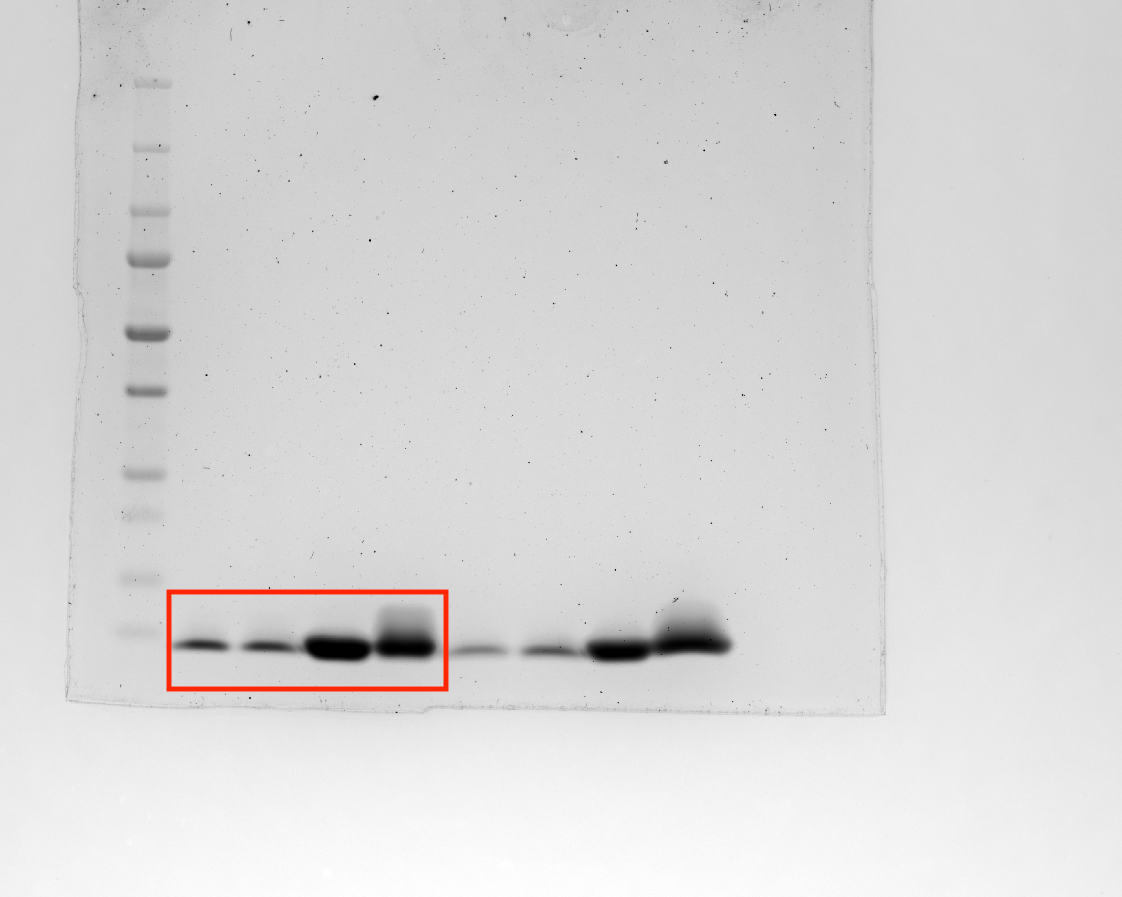

Supplement: Supplementary file 4 — Source data Fig. 2 [file 44318_2025_577_MOESM4_ESM.zip › Figure 2/2C/silver stain.tif]

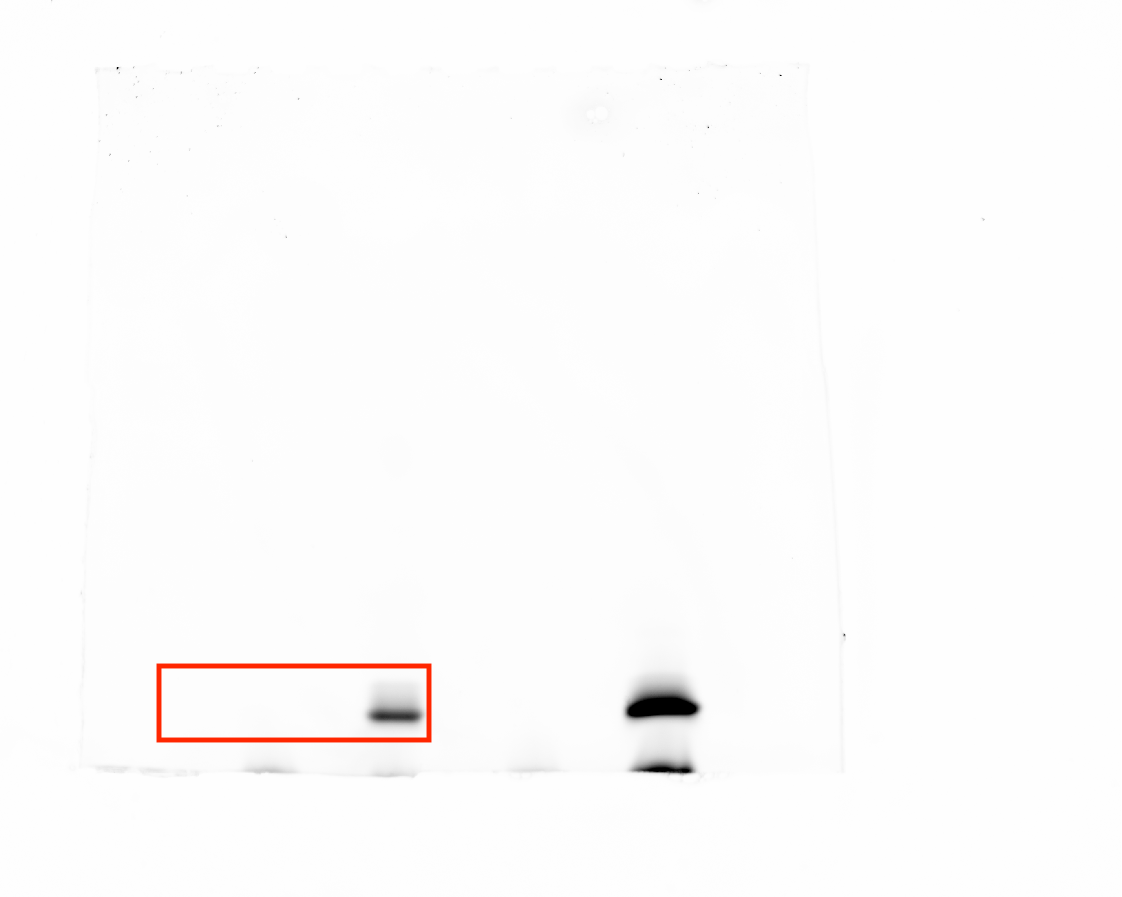

Supplement: Supplementary file 4 — Source data Fig. 2 [file 44318_2025_577_MOESM4_ESM.zip › Figure 2/2C/fluorescence scan.tif]

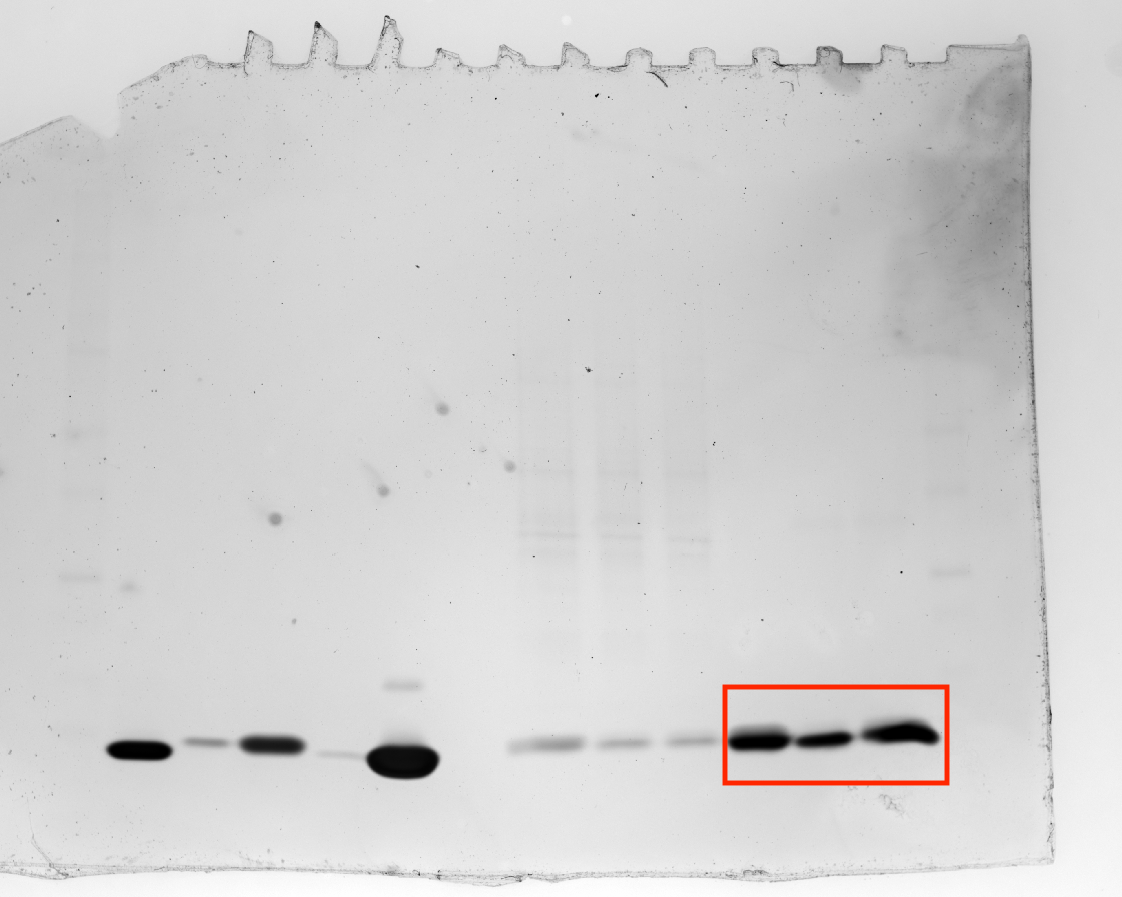

Supplement: Supplementary file 4 — Source data Fig. 2 [file 44318_2025_577_MOESM4_ESM.zip › Figure 2/2D/silver stain.tif]

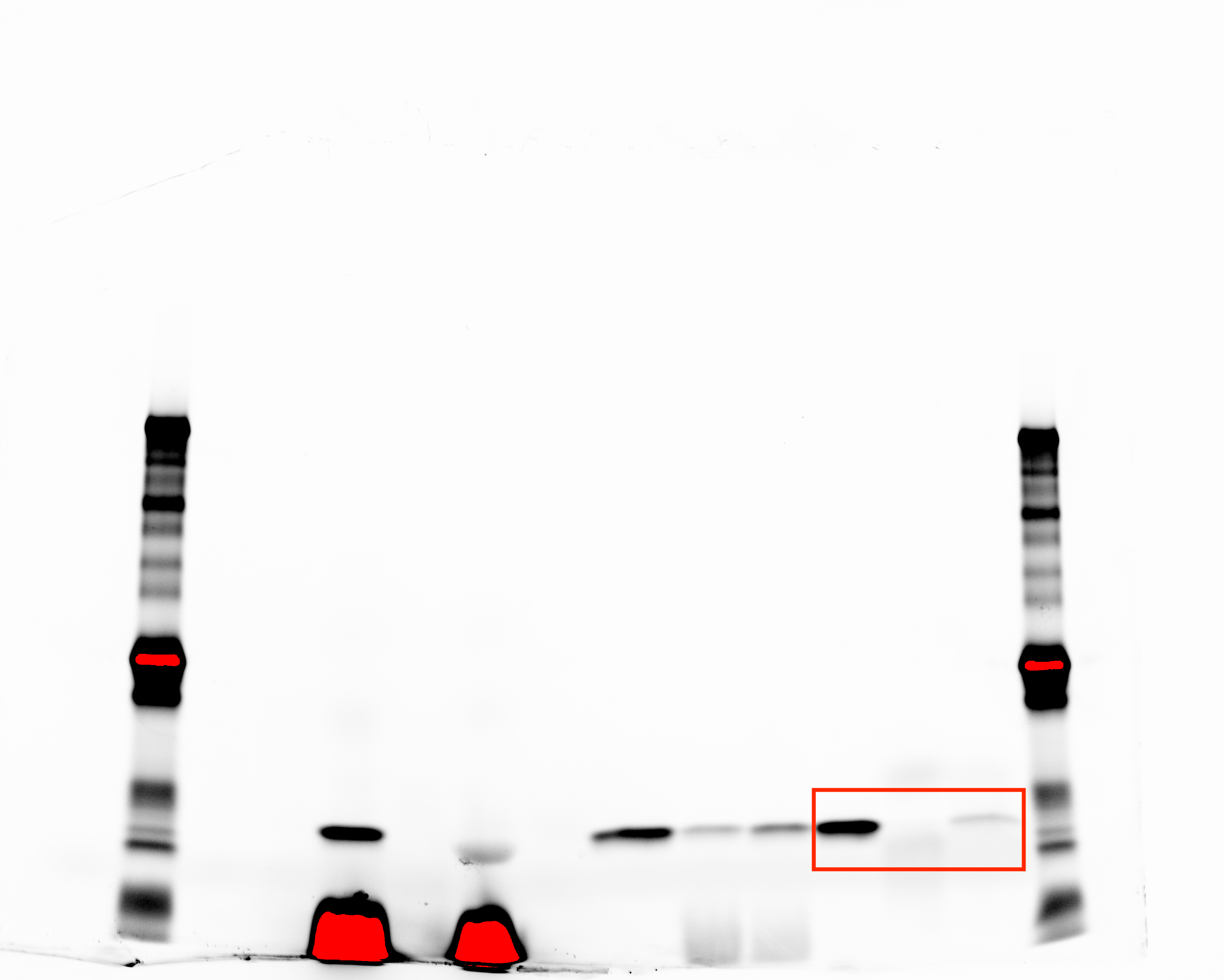

Supplement: Supplementary file 4 — Source data Fig. 2 [file 44318_2025_577_MOESM4_ESM.zip › Figure 2/2D/fluorescence scan.tif]

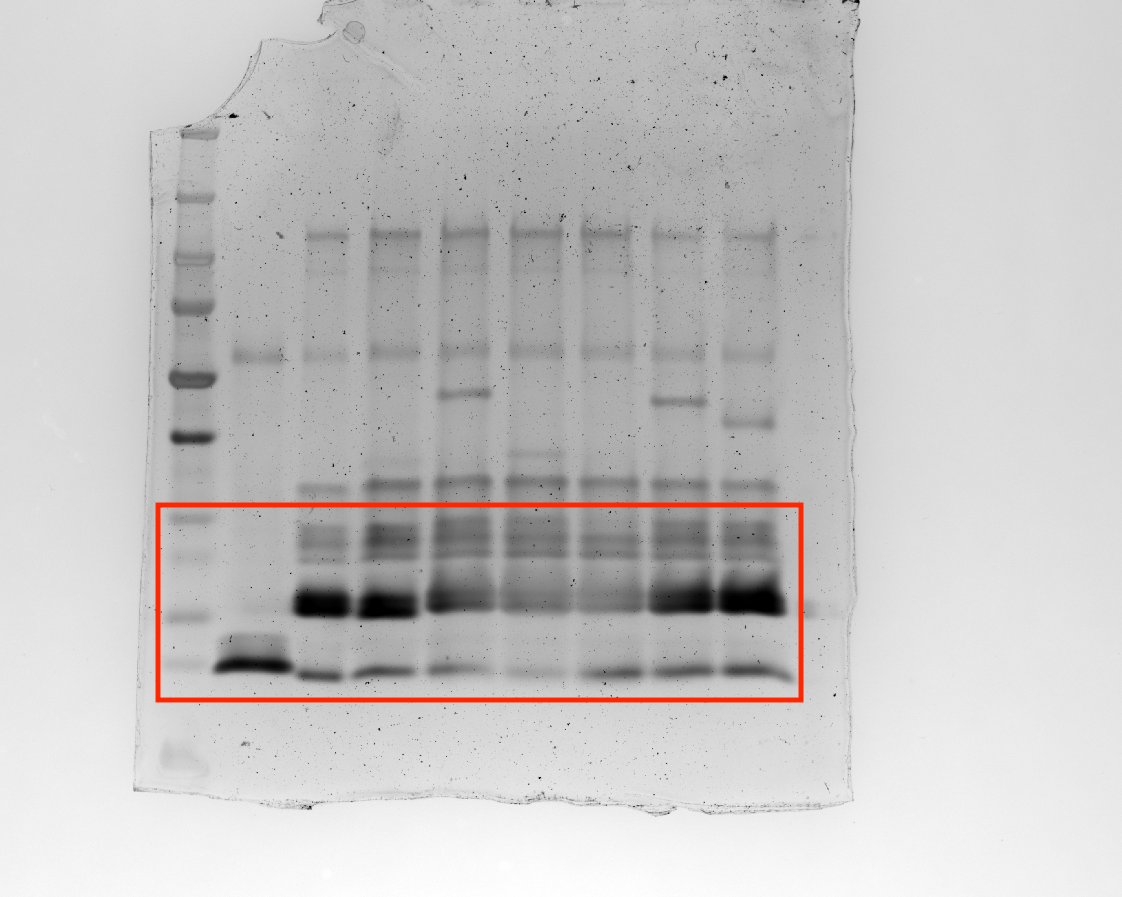

Supplement: Supplementary file 6 — Source data Fig. 4 [file 44318_2025_577_MOESM6_ESM.zip › Figure 4/4C/silver stain.tif]

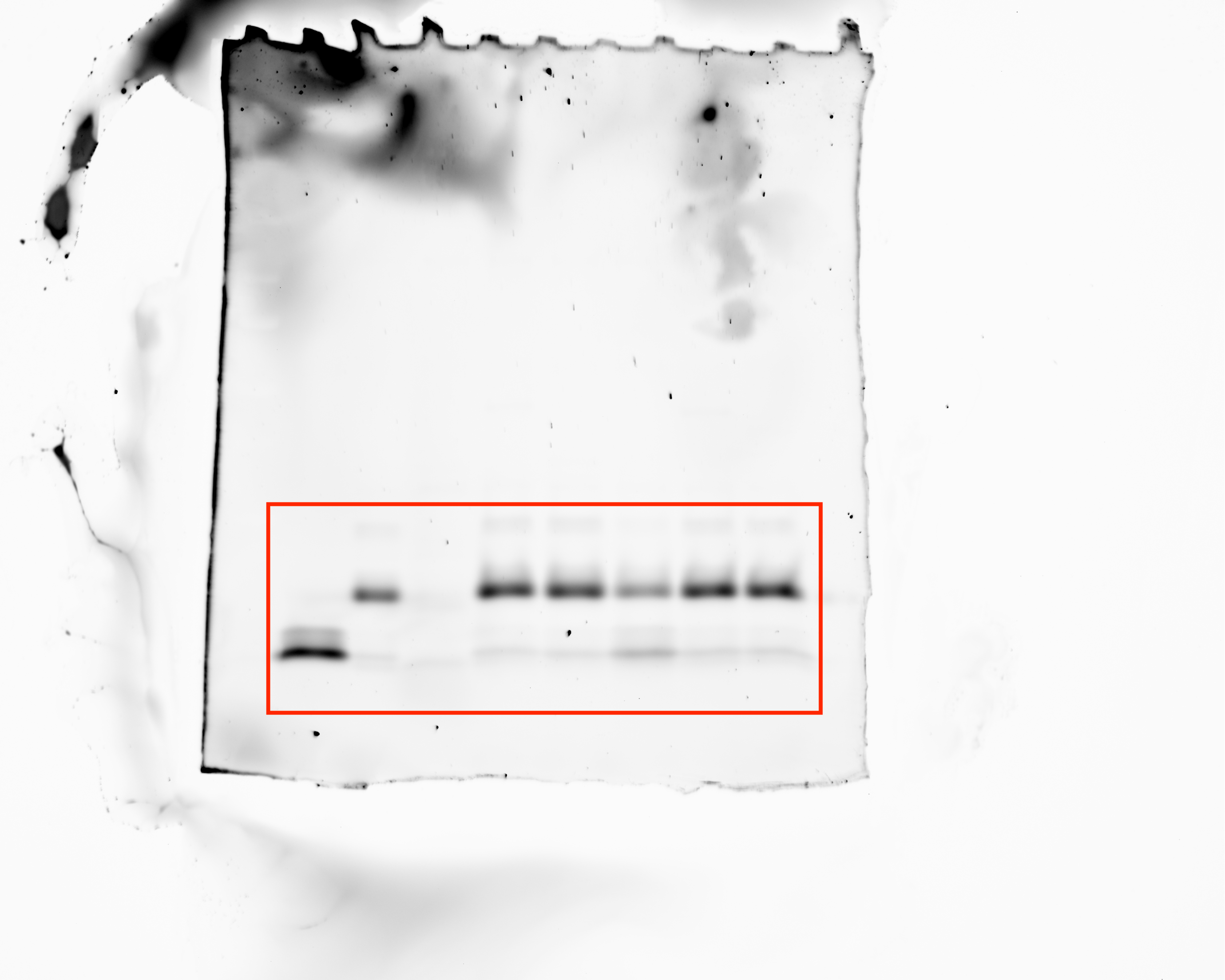

Supplement: Supplementary file 6 — Source data Fig. 4 [file 44318_2025_577_MOESM6_ESM.zip › Figure 4/4C/fluorescence scan.tif]

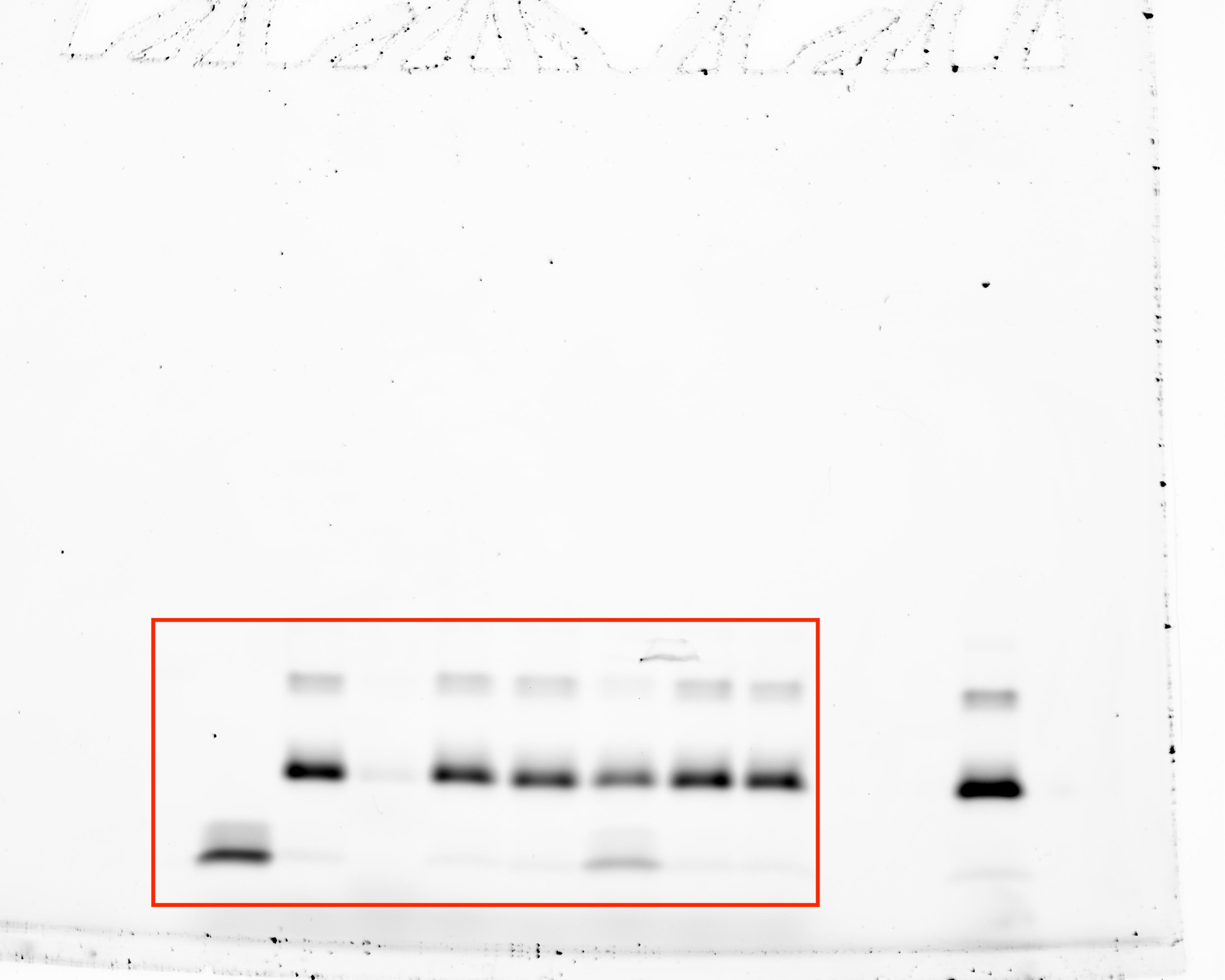

Supplement: Supplementary file 7 — Source data Fig. 5 [file 44318_2025_577_MOESM7_ESM.zip › Figure 5/5I/fluorescence scan.tif]
