# Supplementary material for: RNF114 and RNF166 exemplify reader-writer E3 ligases that extend K11 polyubiquitin onto sites of MARUbylation
Source: EMBO J. 2025 Oct 2;44(21):5993–6018. doi: 10.1038/s44318-025-00577-z (PMC12583694; doi:10.1038/s44318-025-00577-z)
Supplement: Supplementary file 5 — Source data Fig. 3 [file 44318_2025_577_MOESM5_ESM.zip › Figure 3/3A-D/RNF114-consurf_1741725099/consurf_colored_seq_CBS.pdf]

ConSurf Results for job:114-FL date:11/03/2025

```
1                    11                   21                   31                   41
M A A Q Q R D C G G  A A Q L A G P A A E  A D P L G R F T C P  V C L E V Y E K P V  Q V P C G H V F C S
e e e e e e e e e e  e e e e e e e e e e  e e e e e e b e b e  b b b e b b e e e b  e b e b e e e b b e
  f                                                       s      s             f            f   s s

51                   61                   71                   81                   91
A C L Q E C L K P K  K P V C G V C R S A  L A P G V R A V E L  E R Q I E S T E T S  C H G C R K N F F L
e b b e e b e e e e  e e e b e e b e e e  b e e e e e b e e b  e e e b e e e e e e  b e e e e e e b b b
  s                    f   s f f s f                                                s     f

101                  111                  121                  131                  141
S K I R S H V A T C  S K Y Q N Y I M E G  V K A T I K D A S L  Q P R N V P N R Y T  F P C P Y C P E K N
e e b e e e b e e b  e e b e e e b e e e  b e e e e e e e e e  e e e e e e e e b e  b e b e e e e e e e
          f       s                                                      f f f   f  s   s f   f       f

151                  161                  171                  181                  191
F D Q E G L V E H C  K L F H S T D T K S  V V C P I C A S M P  W G D P N Y R S A N  F R E H I Q R R H R
e e e e e b e e e b  e e e e e e e e e e  b b b b b b b e b b  e e e e e e e b e e  b b e e b e e e e e
  f             f s        f              s s   s   s   f s s  f f f f       s   f  s     f       f f

201                  211                  221
F S Y D T F V D Y D  V D E E D M M N Q V  L Q R S I I D Q
b e e e b b b e b e  e e e e e b b e e b  b e e e e e e e
s f f f s   s f        f f                    f f     f
```

The conservation scale:

1 2 3 4 5 6 7 8 9

Variable   Average   Conserved

e - An exposed residue according to the neural network algorithm.

b - A buried residue according to the neural network algorithm.

f - A predicted functional residue (highly conserved and exposed).

s - A predicted structural residue (highly conserved and buried).
