# Supplementary material for: RNF114 and RNF166 exemplify reader-writer E3 ligases that extend K11 polyubiquitin onto sites of MARUbylation
Source: EMBO J. 2025 Oct 2;44(21):5993–6018. doi: 10.1038/s44318-025-00577-z (PMC12583694; doi:10.1038/s44318-025-00577-z)
Supplement: Supplementary file 7 — Source data Fig. 5 [file 44318_2025_577_MOESM7_ESM.zip › Figure 5/5C/RNF125-consurf_1746159990/consurf_colored_seq.pdf]

ConSurf Results for job:RNF125 date:02/05/2025

```
1           11          21          31          41
MGSVLSTDSG  KSAPASATAR  ALERRRDPEL  PVTSFDCAVC  LEVLHQPVRT
eeeebeeeee  eeeeeeebee  eeeeeeeeee  eeeebebbbb  bebbeeebee
fff  f                                    s      f    f  f

51          61          71          81          91
RCGHVFCRSC  IATSLKNNKW  TCPYCRAYLP  SEGVPATDVA  KRMKSEYKNC
ebeebbbebb  beebbeeeee  ebeebeeebe  eeebebbebe  eebeeeeeeb
  ff ss  s     s         sffsf       f   s f      s      s

101         111         121         131         141
AECDTLVCLS  EMRAHIRTCQ  KYIDKYGPLQ  ELEETAARCV  CPFCQRELYE
eeeeeebbbe  ebeeebeebe  eebeeeeeee  eeeeeeeebe  beebeeeeee
 ff      f   sf f        f     f           f    sf s

151         161         171         181         191
DSLLDHCITH  HRSERRPVFC  PLCRLIPDEN  PSSFSGSLIR  HLQVSHTLFY
eebbeebbeb  eeeeeeebbb  bbbebeeeee  eeeeeeebbe  bbebebebbb
     f      f  f   s s        f                 s    s

201         211         221         231
DDFIDFNIIE  EALIRRVLDR  SLLEYVNHSN  TT
eebbebebbe  ebbbeebbee  bbbebbeeee  ee
 f       f              s            f
```

The conservation scale:

1	2	3	4	5	6	7	8	9

Variable — Average — Conserved

e - An exposed residue according to the neural network algorithm.

b - A buried residue according to the neural network algorithm.

f - A predicted functional residue (highly conserved and exposed).

s - A predicted structural residue (highly conserved and buried).
