# Supplementary material for: RNF114 and RNF166 exemplify reader-writer E3 ligases that extend K11 polyubiquitin onto sites of MARUbylation
Source: EMBO J. 2025 Oct 2;44(21):5993–6018. doi: 10.1038/s44318-025-00577-z (PMC12583694; doi:10.1038/s44318-025-00577-z)
Supplement: Supplementary file 7 — Source data Fig. 5 [file 44318_2025_577_MOESM7_ESM.zip › Figure 5/5C/RNF138-consurf_1746160068/consurf_colored_seq_CBS.pdf]

ConSurf Results for job:RNF138 date:02/05/2025

```
1          11         21         31         41
MAEDLSAATS YTEDDFYCPV CQEVLKTPVR TTACQHVFCR KCFLTAMRES
eeeeeeebee eeeeebebeb beebbeeebe eeebeebbbe ebbbebbeee
                  sf     s   f   f    f     s   s

51         61         71         81         91
GAHCPLCRGN VTRRERACPE RALDLENIMR KFSGSCRCCA KQIKFYRMRH
eeebeebeee beeeeeeeee ebeebeebbe eeeeebebee eebebbebee
    f sff      f       s f    s        f f         sf

101        111        121        131        141
HYKSCKKYQD EYGVSSIIPN FQISQDSVGN SNRSETSTSD NTETYQENTS
ebeebeeeee eeeeeeebee beeeeeeeee eeeeeeeeee eeeeeeeeee
f      f f f f

151        161        171        181        191
SSGHPTFKCP LCQESNFTRQ RLLDHCNSNH LFQIVPVTCP ICVSLPWGDP
eeeeeebebe ebeeeeeeee ebbeebeeee beebbebbbb bbbbbeeeee
         f         f   s  fs   f             s   f

201        211        221        231        241
SQITRNFVSH LNQRHQFDYG EFVNLQLDEE TQYQTAVEES FQVNI
eebbeebbbe bbeeeebeee ebbebeeeee eebeebbeee beeee
   s f   f    ff   f         ff           f    f
```

The conservation scale:

1	2	3	4	5	6	7	8	9

Variable — Average — Conserved

e - An exposed residue according to the neural network algorithm.

b - A buried residue according to the neural network algorithm.

f - A predicted functional residue (highly conserved and exposed).

s - A predicted structural residue (highly conserved and buried).
