# Supplementary material for: RNF114 and RNF166 exemplify reader-writer E3 ligases that extend K11 polyubiquitin onto sites of MARUbylation
Source: EMBO J. 2025 Oct 2;44(21):5993–6018. doi: 10.1038/s44318-025-00577-z (PMC12583694; doi:10.1038/s44318-025-00577-z)
Supplement: Supplementary file 7 — Source data Fig. 5 [file 44318_2025_577_MOESM7_ESM.zip › Figure 5/5C/RNF138-consurf_1746160068/consurf_colored_seq.pdf]

ConSurf Results for job:RNF138 date:02/05/2025

```
1
M A E D L S A A T S
e e e e e e e b e e

11
Y T E D D F Y C P V
e e e e e b e b e b
              s f

21
C Q E V L K T P V R
b e e b b e e e b e
      s       f

31
T T A C Q H V F C R
e e e b e e b b b e
f         f

41
K C F L T A M R E S
e b b b e b b e e e
  s       s

51
G A H C P L C R G N
e e e b e e b e e e
        f   s f f

61
V T R R E R A C P E
b e e e e e e e e e
        f

71
R A L D L E N I M R
e b e e b e e b b e
  s   f         s

81
K F S G S C R C C A
e e e e e b e b e e
            f   f

91
K Q I K F Y R M R H
e e b e b b e b e e
              s f

101
H Y K S C K K Y Q D
e b e e b e e e e e
f             f   f

111
E Y G V S S I I P N
e e e e e e e b e e
f   f

121
F Q I S Q D S V G N
b e e e e e e e e e

131
S N R S E T S T S D
e e e e e e e e e e

141
N T E T Y Q E N T S
e e e e e e e e e e

151
S S G H P T F K C P
e e e e e e b e b e
                  f

161
L C Q E S N F T R Q
e b e e e e e e e e
                f

171
R L L D H C N S N H
e b b e e b e e e e
  s     f s       f

181
L F Q I V P V T C P
b e e b b e b b b b

191
I C V S L P W G D P
b b b b b e e e e e
  s       f

201
S Q I T R N F V S H
e e b b e e b b b e
      s   f       f

211
L N Q R H Q F D Y G
b b e e e e b e e e
      f f       f

221
E F V N L Q L D E E
e b b e b e e e e e
              f f

231
T Q Y Q T A V E E S
e e b e e b b e e e
                  f

241
F Q V N I
b e e e e
      f
```

The conservation scale:

1 2 3 4 5 6 7 8 9

Variable **Average** **Conserved**

e - **An exposed residue according to the neural network algorithm.**

b - **A buried residue according to the neural network algorithm.**

f - **A predicted functional residue (highly conserved and exposed).**

s - **A predicted structural residue (highly conserved and buried).**
