# Supplementary material for: RNF114 and RNF166 exemplify reader-writer E3 ligases that extend K11 polyubiquitin onto sites of MARUbylation
Source: EMBO J. 2025 Oct 2;44(21):5993–6018. doi: 10.1038/s44318-025-00577-z (PMC12583694; doi:10.1038/s44318-025-00577-z)
Supplement: Supplementary file 7 — Source data Fig. 5 [file 44318_2025_577_MOESM7_ESM.zip › Figure 5/5C/RNF166-consurf_1746160093/consurf_colored_seq_CBS.pdf]

ConSurf Results for job:RNF166 date:02/05/2025

```
1          11         21         31         41
MAMFRSLVAS AQQRQPPAGP AGGDSGLEAQ YTCPICLEVY HRPVAIGSCG
eebbebbbee eeeeeeeeee eeeeeeeeee bebebbbebb eeebebbebe
ff sf                              sf s f     f     s 

51         61         71         81         91
HTFCGECLQP CLQVPSPLCP LCRLPFDPKK VDKATHVEKQ LSSYKAPCRG
eebbeebbee beeeeeeebe ebeeeeeeee eeebeebeee beeeeeebee
f  s  s          f sf  sf                          s  

101        111        121        131        141
CNKKVTLAKM RVHISSCLKV QEQMANCPKF VPVVPTSQPI PSNIPNRSTF
eeeebbbeeb eeebeebeeb eeebeebeeb eeeeeeeeee eeeeeeebeb
f          f f   s                              fff fs

151        161        171        181        191
ACPYCGARNL DQQELVKHCV ESHRSDPNRV VCPICSAMPW GDPSYKSANF
ebeeeeeeee eeeebeeebe eeeeeeeeeb bbbbbbebbe eeeeeebeeb
 sf f   f  f   s  f     f        s s s fssf ff    s fs

201        211        221        231
LQHLLHRHKF SYDTFVDYSI DEEAAFQAAL ALSLSEN
beebeeeeeb eeebbbebee eeeebbeebb eeeeeee
  f   ff s fffs sf    ff      s   ff   f
```

The conservation scale:

1	2	3	4	5	6	7	8	9

Variable **Average** **Conserved**

e - An exposed residue according to the neural network algorithm.
b - A buried residue according to the neural network algorithm.
f - A predicted functional residue (highly conserved and exposed).
s - A predicted structural residue (highly conserved and buried).
