# Supplementary material for: RNF114 and RNF166 exemplify reader-writer E3 ligases that extend K11 polyubiquitin onto sites of MARUbylation
Source: EMBO J. 2025 Oct 2;44(21):5993–6018. doi: 10.1038/s44318-025-00577-z (PMC12583694; doi:10.1038/s44318-025-00577-z)
Supplement: Supplementary file 8 — Expanded View Figures [file 44318_2025_577_MOESM8_ESM.pdf]

Expanded View Figures

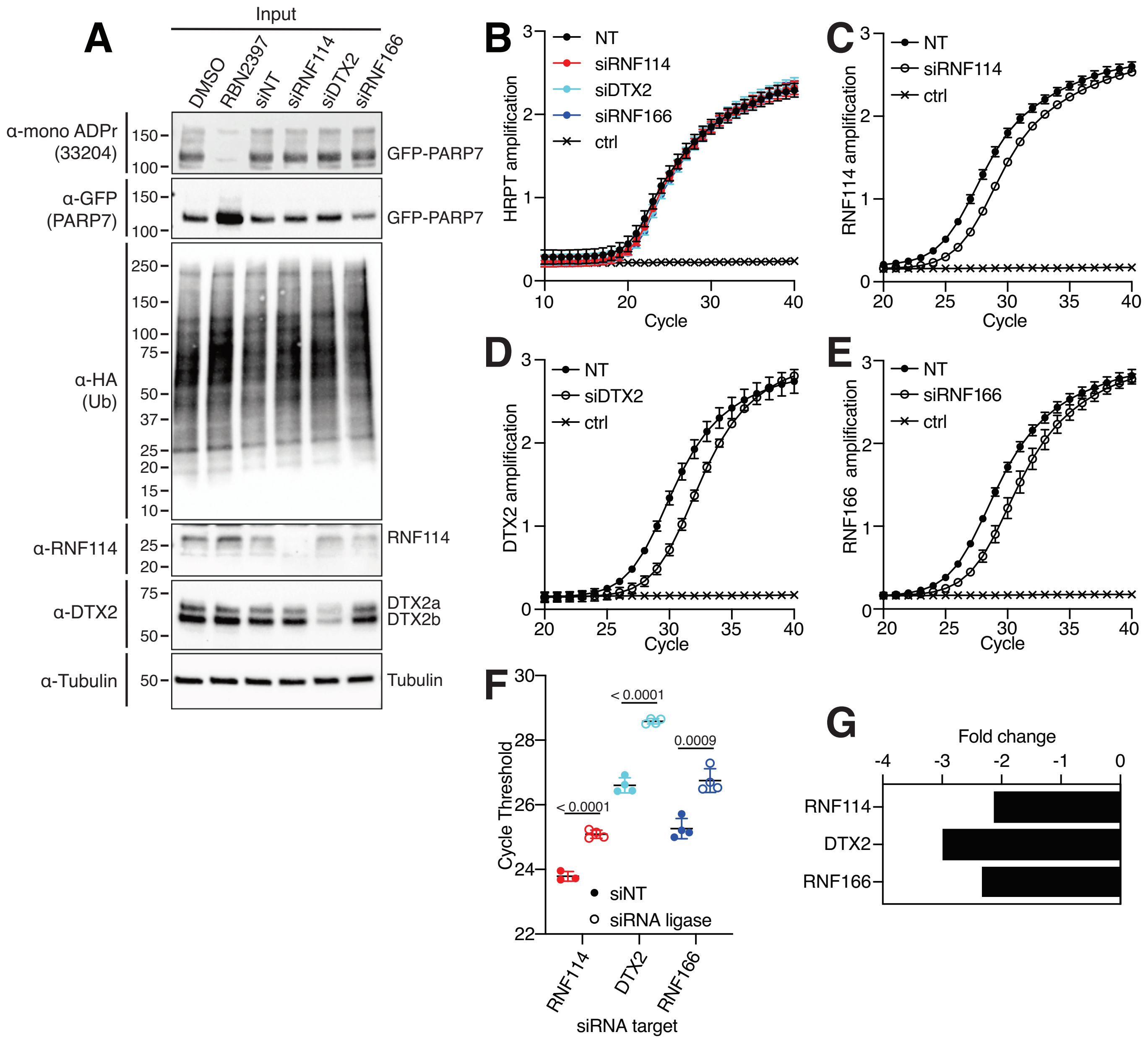

Figure EV1. RNF114 extends PARP7 MARUbylation.

(**A**) Input blots corresponding to Fig. 1C prior to the MARUbylation assay to separate PARP7-bound canonical ubiquitylation from MARUbylation. Genetic knockdown was further confirmed using reverse transcription quantitative PCR (RT-qPCR) and the amplification curves are shown for (**B**) housekeeping gene HRPT, (**C**) RNF114, (**D**) DTX2, and (**E**) RNF166. In these panels, the ctrl refers to a no cDNA control generated from the siNT transfected cells. Data show the mean ± standard deviation for $n = 3$ technical replicates. (**F**) The cycle threshold for amplification of the indicated ligases are shown for the siNT treatment and the respective siE3 treatment. The mean ± standard deviation cycle threshold for $n = 4$ technical replicates for each siE3 treatment was compared to that for the siNT treatment. Significance was assessed using an unpaired *t* test and *P* values are shown on the graph where ***0.001 > *P* > 0.0001, ****$P < 0.0001$. (**G**) Fold change in gene expression was calculated using the delta-delta Ct method.

© The Author(s)

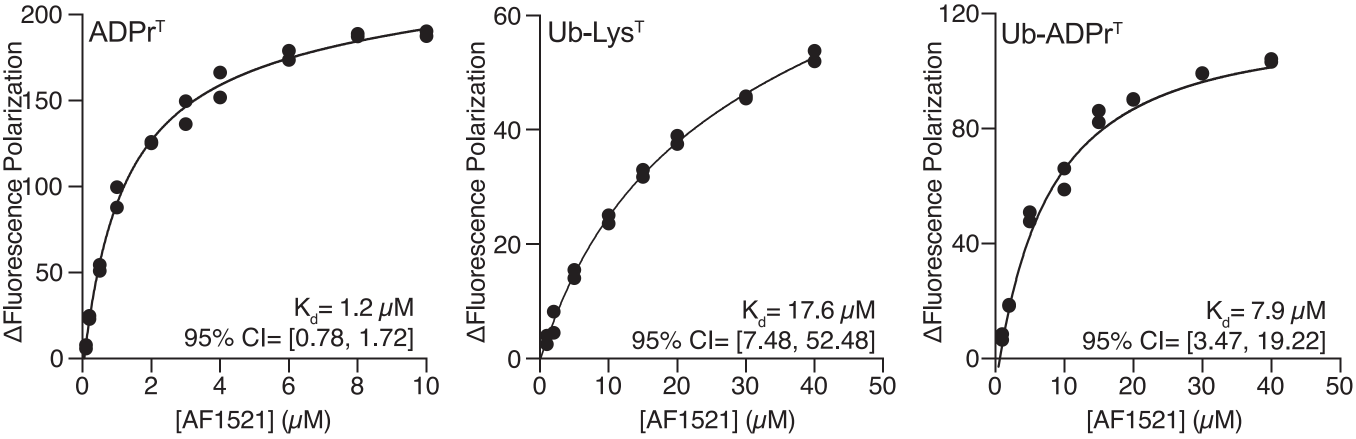

Figure EV2. Validation of fluorescent $ADPr^T$.

Fluorescence polarization binding experiment of $ADPr^T$, Ub-Lys^T, and Ub-$ADPr^T$ with the AF1521 macrodomain to validate the substrates against a known ADPr-binding protein. K_d values and the associated 95% confidence intervals (CI) were derived using GraphPad Prism 10 using a one-site total binding fit.

© The Author(s)

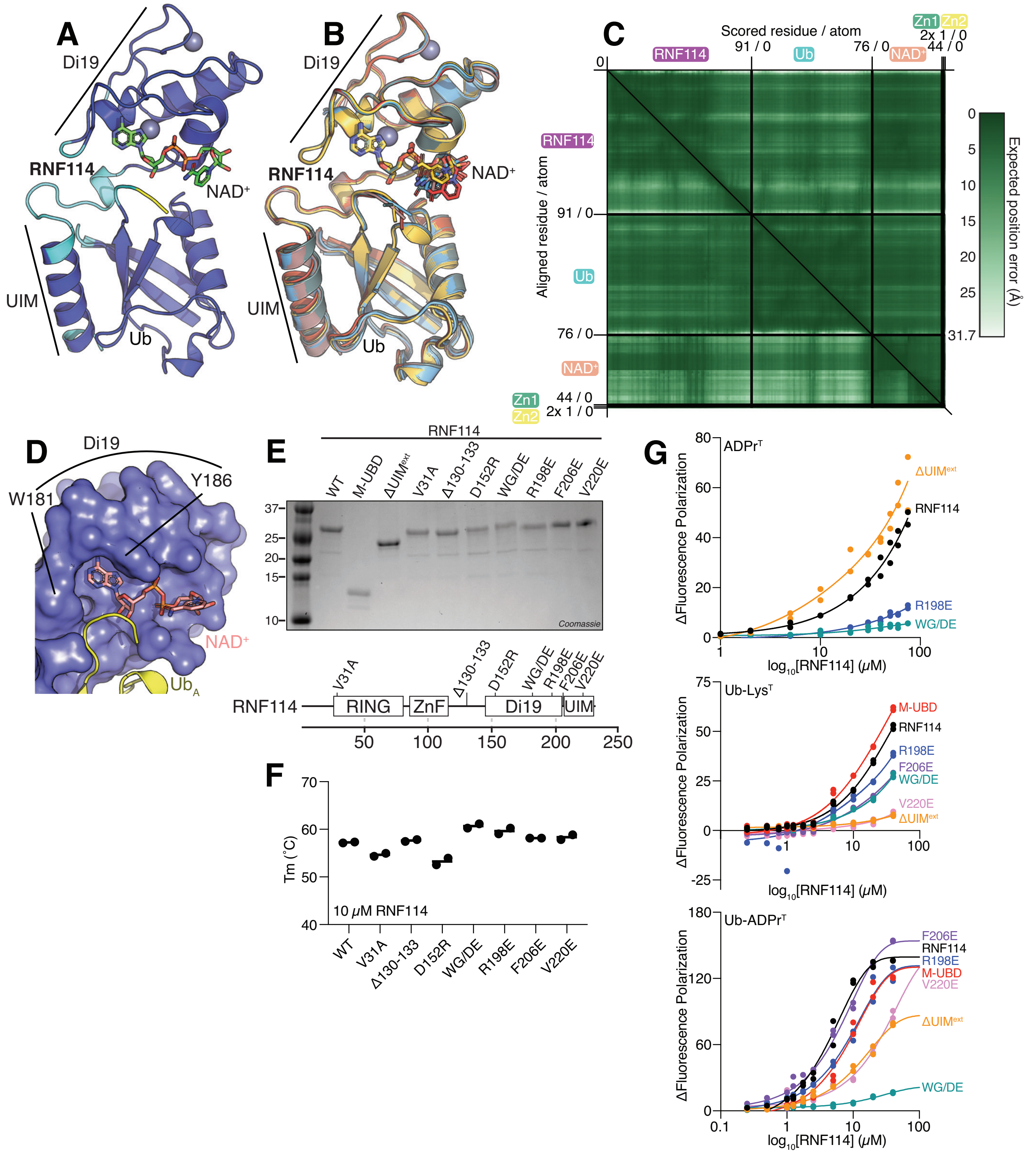
A
Di19
RNF114
NAD+
UIM
Ub
B
Di19
RNF114
NAD+
UIM
Ub
C
Scored residue / atom
RNF114
Ub
NAD+
Zn1
Zn2
Aligned residue / atom
Expected position error (Å)
D
Di19
W181
Y186
NAD+
UbA
E
RNF114
WT
M-UBD
ΔUIMext
V31A
Δ130-133
D152R
WG/DE
R198E
F206E
V220E
Coomassie
RING
ZnF
Di19
UIM
F
Tm (°C)
10 μM RNF114
G
ADPrT
ΔFluorescence Polarization
log10[RNF114] (μM)
Ub-LysT
Ub-ADPrT

Figure EV3. RNF114 binds NAD$^+$ and Ub.

(**A**) The M-UBD of RNF114 was modeled in AlphaFold3 with two Zn^{2+} ions, NAD$^+$, and Ub. The cartoon diagram of the complex is shown with NAD$^+$ in green and protein colored by AlphaFold3 confidence (pLDDT) where blue represents pLDDT > 90, cyan 70 > pLDDT > 90, yellow 50 > pLDDT > 70, and orange pLDDT < 50. The Di19 domain and UIM that make up the M-UBD of RNF114 are labeled. (**B**) Superimposition of the top 5 AlphaFold3 models of the M-UBD:NAD$^+$:Ub complex in different colors. (**C**) PAE plot from AlphaFold3 of the M-UBD:NAD$^+$:Ub model shown in (**A**). Darker green indicates higher confidence in the model due to a lower expected position error. (**D**) W181 and Y186 contribute to the positioning of NAD$^+$ in the binding pocket. Surface representation of the RNF114 Di19 domain accentuates the pocket that accommodates the adenine ring of NAD$^+$. (**E**) RNF114 mutants used in this study were diluted to 1 µM and visualized by SDS-PAGE and Coomassie staining. A schematic of RNF114 and the location of the chosen mutations is also shown. (**F**) Melting temperatures of RNF114 and its mutants derived from a thermal stability assay. (**G**) Fluorescence polarization binding curves for the indicated RNF114 mutants with either ADPr^T, Ub-Lys^T, or Ub-ADPr^T as shown in Fig. 3E instead showing the RNF114 concentration (x-axis) on a log scale for ease of comparison.

© The Author(s)

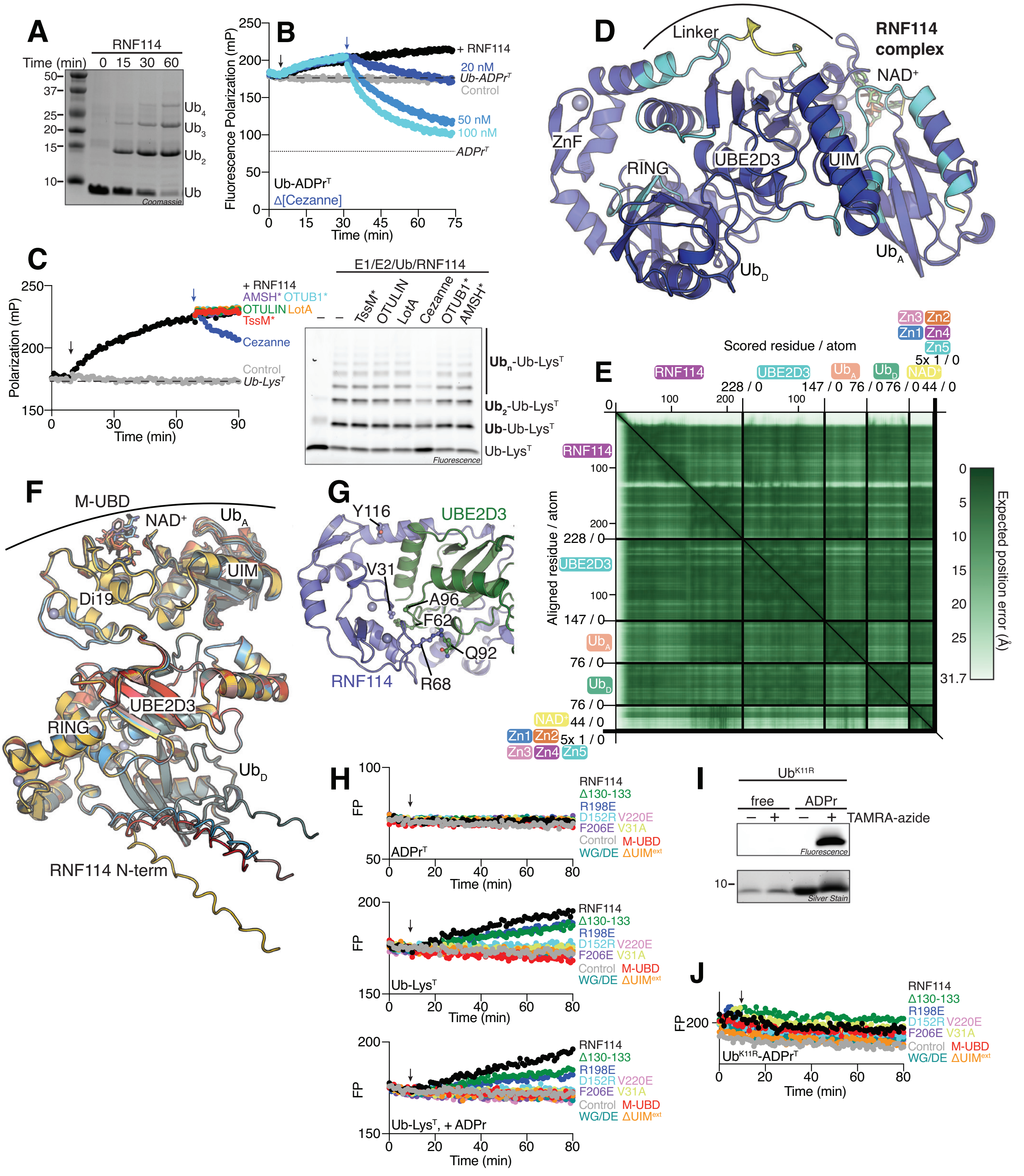
A
RNF114
Time (min) 0 15 30 60
Ub4
Ub3
Ub2
Ub
Coomassie
B
+ RNF114
20 nM
Ub-ADPr
Control
50 nM
100 nM
ADPr
Ub-ADPr
Δ[Cezanne]
Fluorescence Polarization (mP)
Time (min)
C
+ RNF114
AMSH* OTUB1*
OTULIN LotA
TssM*
Cezanne
Control
Ub-Lys
Polarization (mP)
Time (min)
E1/E2/Ub/RNF114
TssM* OTULIN LotA Cezanne OTUB1* AMSH*
Ubn-Ub-Lys
Ub2-Ub-Lys
Ub-Ub-Lys
Ub-Lys
Fluorescence
D
Linker
RNF114 complex
NAD+
ZnF
RING
UBE2D3
UIM
UbA
UbD
E
Scored residue / atom
Aligned residue / atom
Expected position error (Å)
RNF114 UBE2D3 UbA UbD NAD+
Zn1 Zn2 Zn3 Zn4 Zn5
F
M-UBD
NAD+
UbA
UIM
Di19
UBE2D3
RING
UbD
RNF114 N-term
G
Y116
UBE2D3
V31
A96
F62
Q92
R68
RNF114
H
FP
ADPr
Ub-Lys
Ub-Lys, + ADPr
Time (min)
RNF114
Δ130-133
R198E
D152R V220E
F206E V31A
Control M-UBD
WG/DE ΔUIMext
I
UbK11R
free ADPr
TAMRA-azide
Fluorescence
Silver Stain
J
FP
UbK11R-ADPr
Time (min)

Figure EV4. RNF114 catalyzes K11-linked extension on Ub-Lys.

(**A**) RNF114 autoubiquitylation with WT Ub. Samples were removed at the indicated timepoints and quenched in 3X sample buffer prior to separation by SDS-PAGE and Coomassie staining. (**B**) UbiCRest experiment optimizing the concentration of Cezanne to target the K11 isopeptide linkage preferentially over the Ub-ADPr ester linkage. This experiment was conducted as described in the Materials and Methods with the indicated concentrations of Cezanne to identify 20 nM as the optimal Cezanne concentration for our experiments. (**C**) UbiCRest experiment using the indicated deubiquitylases for RNF114 ubiquitylation of Ub-Lys^{T}. The reaction was monitored by fluorescence polarization and the sample was removed from the plate at 90 min, run on SDS-PAGE, and visualized by in-gel fluorescence of the TAMRA fluorophore. (**D**) AlphaFold3 model of the full-length RNF114, NAD^{+}, UBE2D3, two copies of Ub, and five Zn^{2+} ions. The cartoon diagram of the complex is shown with NAD^{+} in green and protein colored by AlphaFold3 confidence (pLDDT) where blue represents pLDDT > 90, cyan 70 > pLDDT > 90, yellow 50 > pLDDT > 70, and orange pLDDT <50. The RING and ZnF domains and UIM of RNF114, UBE2D3, Ub_{D}, the UIM-bound Ub (Ub_{A}), in addition to the linker of RNF114 that mediates backside UBE2D3 binding are labeled. (**E**) PAE plot from AlphaFold3 for the RNF114 transferase complex shown in (**D**). Darker green indicates higher confidence in the model due to a lower expected position error. (**F**) The top 5 AlphaFold3 models from (**D**) are shown aligned and colored differently. Each protein and specific regions of RNF114 are labeled. (**G**) Detailed view of the modeled interface between the RNF114 RING domain and UBE2D3. V31 and the linchpin residue R68 are shown from the RING domain of RNF114 and their proximity to F62/A96 and Q92, respectively. Y116, a tyrosine phosphorylation site, is also shown in this view. (**H**) UbiReal curves showing the activity of RNF114 mutants against the ADPr^{T} and Ub-Lys^{T} ± unlabeled ADPr substrates. These graphs show a representative dataset of $n = 2$ technical replicates. (**I**) Validation of the Ub^{K11R}-ADPr^{T} substrate (products of the CuAAC reaction) by SDS-PAGE and in-gel fluorescence. (**J**) UbiReal experiment for RNF114 mutants against the Ub^{K11R}-ADPr^{T} substrate. For all fluorescence polarization panels, the black arrow represents the timepoint when ATP was added to the reaction to initiate RNF114 ligase activity, and the blue arrow represents the timepoint when the indicated deubiquitylase was added to cleave the products.

 © The Author(s)

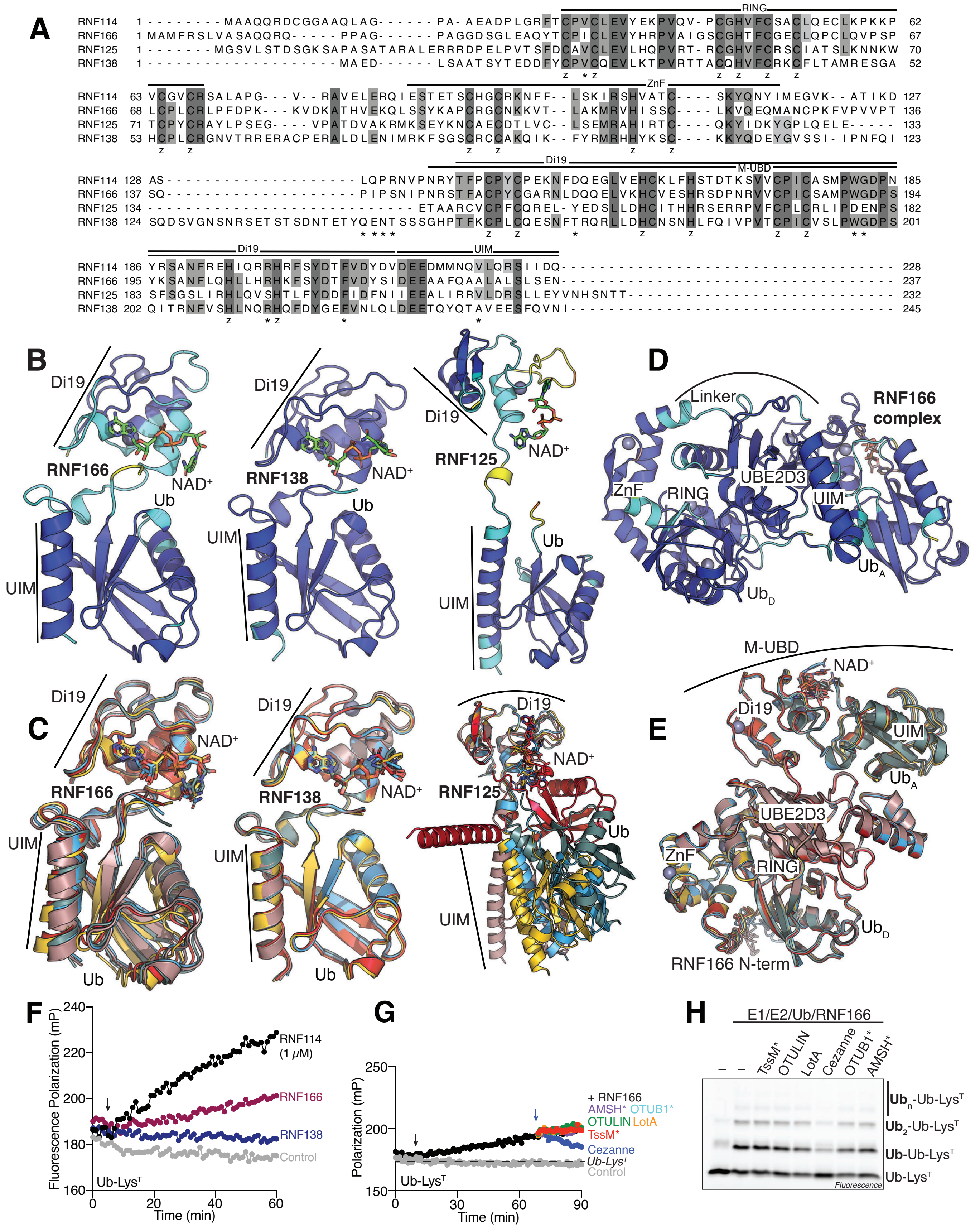
A
RING
RNF114 1 --------MAAQQRDCGGAAQLAG------PA-AEADPLGRFTCPVCLEVYEKPVQV-PCGHVFCSACLQECLKPKKP 62
RNF166 1 MAMFRSLVASAQQRQ-----PPAG------PAGGDSGLEAQYTCPICLEVYHRPVAIGSCGHTFCGECLQPCLQVPSP 67
RNF125 1 -------MGSVLSTDSGKSAPASATARALERRRDPELPVTSFDCAVCLEVLHQPVRT-RCGHVFCRSCIATSLKNNKW 70
RNF138 1 -------------------MAED------LSAATSYTEDDFYCPVCQEVLKTPVRTTACQHVFCRKCFLTAMRESGA 52
ZnF
RNF114 63 VCGVCRSALAPG---V--RAVELERQIESTETSCHGCRKNFF--LSKIRSHVATC----SKYQNYIMEGVK--ATIKD 127
RNF166 68 LCPLCRLPFDPK---KVDKATHVEKQLSSYKAPCRGCNKKVT--LAKMRVHISSC----LKVQEQMANCPKFVPVVPT 136
RNF125 71 TCPYCRAYLPSEG----VPATDVAKRMKSEYKNCAECDTLVC--LSEMRAHIRTC----QKYIDKYGPLQELE----- 133
RNF138 53 HCPLCRGNVTRRERACPERALDLENIMRKFSGSCRCCAKQIK--FYRMRHHYKSC----KKYQDEYGVSSI-IPNFQI 123
Di19
M-UBD
RNF114 128 AS-------------------LQPRNVPNRYTFPCPYCPEKNFDQEGLVEHCKLFHSTDTKSVVCPICASMPWGDPN 185
RNF166 137 SQ-------------------PIPSNIPNRSTFACPYCGARNLDQQELVKHCVESHRSDPNRVVCPICSAMPWGDPS 194
RNF125 134 ----------------------ETAARCVCPFCQREL-YEDSLLDHCITHHRSERRPVFCPLCRLIPDENPS 182
RNF138 124 SQDSVGNSNRSETSTSDNTETYQENTSSSGHPTFKCPLCQESNFTRQRLLDHCNSNHLFQIVPVTCPICVSLPWGDPS 201
Di19
UIM
RNF114 186 YRSANFREHIQRRHRFSYDTFVDYDVDEEDMMNQVLQRSIIDQ---- 228
RNF166 195 YKSANFLQHLLHRHKFSYDTFVDYSIDEEAAFQAALALSLSEN---- 237
RNF125 183 SFSGSLIRHLQVSHTLFYDDFIDFNIIEEALIRRVLDRSLLEYVNHSNTT---- 232
RNF138 202 QITRNFVSHLNQRHQFDYGEFVNLQLDEETQYQTAVEESFQVNI---- 245
B
Di19
RNF166
NAD+
Ub
UIM
Di19
RNF138
NAD+
Ub
UIM
Di19
RNF125
NAD+
Ub
UIM
D
Linker
RNF166 complex
ZnF
RING
UBE2D3
UIM
UbA
UbD
C
Di19
NAD+
RNF166
UIM
Ub
Di19
RNF138
NAD+
UIM
Ub
Di19
NAD+
RNF125
Ub
UIM
E
M-UBD
NAD+
Di19
UIM
UbA
UBE2D3
ZnF
RING
UbD
RNF166 N-term
F
Fluorescence Polarization (mP)
RNF114 (1 μM)
RNF166
RNF138
Control
Ub-LysT
Time (min)
G
Polarization (mP)
+ RNF166
AMSH* OTUB1*
OTULIN LotA
TssM*
Cezanne
Ub-LysT
Control
Ub-LysT
Time (min)
H
E1/E2/Ub/RNF166
TssM*
OTULIN
LotA
Cezanne
OTUB1*
AMSH*
Ubn-Ub-LysT
Ub2-Ub-LysT
Ub-Ub-LysT
Ub-LysT
Fluorescence

Figure EV5. Ub-ADPr recognition is conserved in a family of MARUbe-Targeted Ligases (M-UTLs).

(**A**) Sequence alignment of M-UTLs RNF114, RNF166, RNF125, and RNF138. The RING and ZnF domains, M-UBD, Di19, and UIM are labeled based on the sequence of RNF114. Sites of point mutation in our experiments with RNF114 are indicated by asterisks, and Zn^{2+}-coordinating residues are labeled (z). Sequences were aligned using Jalview and colored according to percent identity where the darker the gray corresponds to higher conservation. (**B**) Cartoon diagrams of the M-UBD of RNF166, RNF138, or RNF125 in complex with Ub and NAD^+ colored by the AlphaFold3 confidence scale. (**C**) Alignment of the top 5 models for each M-UBD (RNF166, RNF138, or RNF125) in complex with NAD^+ and Ub. (**D**) AlphaFold3 model of RNF166 in complex with UBE2D3, NAD^+, two copies of Ub, and five Zn^{2+} ions, colored by confidence. (**E**) Overlay of the top 5 AlphaFold3 models for the RNF166 ligase complex. (**F**) Representative UbiReal experiment for the indicated E3 ligases with the Ub-Lys^T substrate. In this experiment, RNF114 was used at 1 µM and the other ligases were used at 5 µM. ATP addition is marked by the black arrow. (**G**) Reaction products of a ligase assay for RNF166 with Ub-Lys^T were subjected to a UbiCRest panel. ATP was added to initiate RNF166 ligase activity at the black arrow, and the indicated deubiquitylase was added at the blue arrow. (**H**) The 90-min timepoint from (**G**) was removed from the plate reader and visualized by SDS-PAGE and in-gel fluorescence. (**B**, **D**) For all AlphaFold3 confidence coloring, blue represents pLDDT >90, cyan 70 > pLDDT >90, yellow 50 > pLDDT >70, and orange pLDDT <50.

 © The Author(s)
